# RBFOX1 Cooperates with MBNL1 to Control Splicing in Muscle, Including Events Altered in Myotonic Dystrophy Type 1

**DOI:** 10.1371/journal.pone.0107324

**Published:** 2014-09-11

**Authors:** Roscoe Klinck, Angélique Fourrier, Philippe Thibault, Johanne Toutant, Mathieu Durand, Elvy Lapointe, Marie-Laure Caillet-Boudin, Nicolas Sergeant, Geneviève Gourdon, Giovanni Meola, Denis Furling, Jack Puymirat, Benoit Chabot

**Affiliations:** 1 Department of Microbiology and Infectiology, Faculty of Medicine and Heath Sciences, Université de Sherbrooke, Sherbrooke, Quebec, Canada; 2 Laboratory of Functional Genomics, Faculty of Medicine and Heath Sciences, Université de Sherbrooke, Sherbrooke, Quebec, Canada; 3 Centre de Recherche du CHUL (Centre Hospitalier Universitaire de Québec), Université Laval, Ste-Foy, Quebec, Canada; 4 Inserm UMR 837, Université Lille Nord de France, IFR114/IMPRT, Lille, France; 5 Inserm U781, Hôpital Necker-EM and Université Paris Descartes-Sorbonne Paris Cité, Institut *Imagine*, Paris, France; 6 Department of Biomedical Sciences for Health, University of Milan, Milan, Italy; 7 UPMC-Université Paris 06, Institute of Myology, Paris, France; Biodonostia Institute, Spain

## Abstract

With the goal of identifying splicing alterations in myotonic dystrophy 1 (DM1) tissues that may yield insights into targets or mechanisms, we have surveyed mis-splicing events in three systems using a RT-PCR screening and validation platform. First, a transgenic mouse model expressing CUG-repeats identified splicing alterations shared with other mouse models of DM1. Second, using cell cultures from human embryonic muscle, we noted that DM1-associated splicing alterations were significantly enriched in cytoskeleton (e.g. *SORBS1*, *TACC2*, *TTN*, *ACTN1* and *DMD*) and channel (e.g. *KCND3* and *TRPM4*) genes. Third, of the splicing alterations occurring in adult DM1 tissues, one produced a dominant negative variant of the splicing regulator RBFOX1. Notably, half of the splicing events controlled by MBNL1 were co-regulated by RBFOX1, and several events in this category were mis-spliced in DM1 tissues. Our results suggest that reduced RBFOX1 activity in DM1 tissues may amplify several of the splicing alterations caused by the deficiency in MBNL1.

## Introduction

Myotonic dystrophy type 1 (DM1) is a multisystem disorder that affects primarily skeletal muscles causing myotonia, muscle weakness and degeneration, but also causes impaired heart function, ocular cataracts and various dysfunctions of the central nervous system. DM1 is caused by the expansion of CTG-trinucleotide repeats in the 3′-untranslated region (UTR) of the *DMPK* gene. The most commonly accepted mechanistic explanation for this disease is that the nuclear accumulation of transcripts containing CUG expansions sequesters the RNA binding protein MBNL1 and stabilizes the CELF family member CUGBP1 through hyperphosphorylation [1]–[6]. The disregulated expression and activity of these RNA binding proteins in DM1 individuals leads to perturbations in the alternative splicing program of key genes, such that many are switched to their embryonic profiles [2], [7]. Among the mis-splicing events that have been documented [8], splicing reversions occurring in the muscle chloride channel CLCN1 [9], [10], the insulin receptor INSR [11] and BIN1 [12] contribute respectively to myotonia, insulin resistance and muscle weakness. Since MBNL1 has also been implicated in transcription and other aspects of RNA biogenesis [13]–[15], and since CUGBP1 can regulate translation [16], [17], other defects in gene expression are expected. Moreover, the CUG repeat expansion may have other effects on gene expression, as suggested by a study in a CTG repeat-expressing mouse that identified changes in the abundance of many extracellular matrix mRNAs [14]. In addition to MBNL1 and CUGBP1, the RNA binding proteins hnRNP H and MBNL2 have also been implicated in DM1 pathogenesis [4], [14], [18]–[21].

While the full spectrum of splicing alterations in DM patients remains to be determined, a variety of model systems have been used to study these alterations and determine the contributions that CUG repeats, MBNL downregulation, and CUGBP1 overexpression have to disease evolution [8]. Modeling trinucleotide repeat instability in transgenic mice has allowed the recapitulation of human splicing defects in a few orthologous murine genes [14], [22], and the replication of some of the muscle phenotypes and histopathology of human DM1 [23]–[25]. Notably, MBNL1 knockout mice display myotonia due to abnormal CLCN1 splicing and develop myopathy, but exhibit no sign of muscle degeneration [26]. On the other hand, induced expression of CUGBP1 in adult skeletal muscle or the heart also mimics DM1 histopathology [27], [28]. Microarray analysis has identified mis-splicing events in the skeletal muscle of the HSA^LR^ mouse (FVB/n strain) that expresses approximately 250 CUG-repeats [14]. Comparing its splicing profile with that of MBNL1 knockout mice revealed that 128 of a total of 172 mis-splicing events were common to both mouse models. Thirty-three of these were validated by RT-PCR and three were confirmed to be mis-spliced in the majority or all human samples tested [14].

To reveal splicing alterations that may be relevant to the DM1 phenotypes, we deployed our RT-PCR screening platform to identify which mis-splicing events documented in the HSA^LR^ and MBNL1 knockout mice (FBV/n strain) were similarly altered in mice (C57BL6/129/OLA/FVB strain) displaying a milder DM1 phenotype. We also used the platform to identify muscle-relevant mis-splices in myoblast cell cultures derived from embryonic and adult DM1 tissues. Because one of the DM1 mis-splicing events identified in adult DM1 tissues occurred in the gene encoding the splicing regulator RBFOX1, we further explored the regulatory interconnections between MBNL1 and RBFOX1, and discovered that these RNA binding proteins cooperate to regulate many muscle-relevant genes, a subset of which are mis-spliced in DM1.

## Materials and Methods

The study has been approved by the Ethics Committee of CRCHU de Quebec (project A12-08-1019). Human tissues and cells were obtained from the MyoBank-CHUQ, which has been approved by the Ethics Committee of the CRCHU de Quebec (project A12-08-1022). Anesthesia of mice was done with 2% isoflurane, CO_2_ followed by cervical dislocation. The protocol was approved by the CRCHU de Quebec institutional Animal Care, the “comité de protection des animaux du CHUQ” (CPAC, protocol No 2103151-1).

### Mouse, human cell lines and tissues

Mice carrying the CUG600 repeats are described in [29]. Mice carrying the CUG1200 repeats are called DMSXL and are described in [30]. Human normal satellite muscle cells were derived from quadriceps muscle biopsy of 41 and 47 year old females. Human fetal normal satellite muscle cells (HFN) were derived from a 15 week old fetus. Human DM1 satellite muscle cells carrying 750 CTG repeats (ST-750), were derived from a 20 week old fetus. Human DM1 satellite muscle cells carrying 1200 CTG (ST-1200) and 3500 (ST-3500), were derived from a 13 and a 15 week old fetus. Human muscle satellite cells were grown in MB-1 medium supplemented with 15% heat-inactivated fetal bovine serum, 5 µg/ml insulin, 0.5 mg/ml BSA, 10 ng/ml epidermal growth factor and 0.39 µg/ml dexamethasone (proliferative medium), as previously described [31], [32]. For human muscle satellite cell differentiation, the cells were subsequently transferred to DMEM supplemented with 0.5% heat-inactivated fetal bovine serum,10 µg/ml insulin and 10 µg/ml apo-transferrin (differentiation medium). All cultures were incubated at 37°C in a humid atmosphere containing 5% CO_2_. Normal and DM1 cells were used between the 4th and 6th passages. The number of passages refers to the total number of passages from the time following the isolation of the initial satellite muscle cell population from the fetus. DM1 skeletal muscle samples were obtained from the left biceps brachii of 3 females aged 44, 45 and 48 years old and two males, aged 31 and 40 years old. All donors had an adult form of the disease. Normal skeletal muscle samples were obtained from 3 males aged 32, 41 and 48 years old and one 41 years old female. Biopsies were done during surgical intervention. All human DM1 muscle cell lines and DM1 tissues were obtained from the Quebec DM1 biobank, following consent from the CHUQ ethical committee

RNA interference assay with siRNAs. The siRNAs used to knockdown the expression of RNA-binding proteins were purchased from IDT (Coralville, Iowa) siRNA target sequences were GACGCAAUAACUUGAUUCAdTdT (*MBNL1*) and GGUCUCGUUCUUUCUUCAUdTdT (*RBFOX1*). siRNAs duplexes carrying dTdT 3**′** overhangs were transfected into cells at a concentration of 100****nM using Lipofectamine 2000 (Invitrogen). RNA was extracted 48 hours post-transfection. Knockdown was validated by evaluating relative expression levels by SYBR green based RT-qPCR as previously described [33], [34]. Primer sequences for target and reference genes are listed in **Table S2, qPCR tab**.

### RT-PCR assays

Our collection of alternative splicing units was derived from the RefSeq database. Sets of primers mapping in the exons flanking all the simple alternative splicing events were designed using Primer3 with default parameters. Total RNA was extracted using TRIzol and quantified using a 2100 Bioanalyzer (Agilent Inc. Santa Clara, CA, USA). A total of 2 µg of RNA was reverse transcribed using a mix of random hexamers and oligo(dT) and the Omniscript reverse transcriptase (Qiagen, Germantown, MD, USA) in a final volume of 20 µl. Twenty ng of cDNA were amplified with 0.2 U/10 µl of HotStarTaq DNA Polymerase (Qiagen) in the buffer provided by the manufacturer, and in the presence of the specific primers (IDT) for each splicing unit (at concentrations ranging from 0.3 to 0.6 µM) and dNTPs. The list of ASEs, oligos, and expected size of RT-PCR products are shown in **Table S2**), and primer locations mapped to the UCSC genome browser can be viewed at http://palace.lgfus.ca/data/related/2073/odgene_/. When more than one ASE was targeted per gene, lowercase letter suffixes were appended to gene names, e.g Dnm1l.a, Dnm1l.b, …, see **Tables S1 and S2** and link above for precise locations. Reactions were carried out in the GeneAmp PCR system 9700 (Applied Biosystems, Foster City, CA, USA). A first cycle of 15 minutes at 95°C was followed by 35 cycles of 30 seconds at 94°C, 30 seconds at 55°C and 1 minute at 72°C. Thermocycling was concluded with an extension step of 10 minutes at 72°C. Visualization and analysis of amplified products were done using the LabChip HT DNA assay on a Caliper LC-90 automated microfluidic station (Caliper, Hopkinton, MA, USA).

## Results

### Splicing defects in mouse expressing CUG-repeats

The transgenic C57BL/6-derived mouse strains express 600 and 1200 CUG-repeats, and display phenotypic traits that are characteristic of DM1 [25], but in a milder form compared to the MBNL1 knockout and HSA^LR^ mice [30]. It was therefore of interest to determine to what extent splicing alterations overlap with the splicing alterations observed in the other CUG-expressing HSA^LR^ mouse model (FVB/n strain) [14].

Total RNA from two muscle sources (tibialis anterior and gastrocnemius) were isolated from three normal mice, two DM1 mice expressing 600 repeats (CUG600) and three DM1 mice expressing 1200 repeats (CUG1200). We used our RT-PCR analysis platform [35]–[37] to interrogate a total of 172 alternative splicing events (ASEs) in genes reported to be susceptible to changes in HSA^LR^ and MBNL knockout mice [14]. Because our RT-PCR approach requires the design of primers on either side of each splicing event, only 58 of the 172 ASEs, representing cassette exons, and alternative 5′ and 3′ splice sites could be designed directly. The remaining reported ASEs, comprised mostly of microarray-identified alternate transcript start and end sites, were not directly accessible by our RT-PCR technique. Nonetheless, we were able to identify and design primers for 114 additional ASEs in regions overlapping these affected transcripts. Maps of the 172 ASEs analyzed showing our primer designs and the regions reported by Du et al. [14] can be viewed at http://palace.lgfus.ca/data/related/2080/odgene_/. RT-PCR amplifications followed by microcapillary electrophoretic separation were designed to detect a long and a short isoform of unambiguous identity. For each ASE, the percent spliced-in (PSI or Ψ) value, defined as the ratio between the concentration of the long isoform over the sum of the short and long isoform concentrations, was computed. When more than two amplification products were observed, the short and long products were selected to maximize their total abundance across the reaction set. To assess the significance of our results, we used a statistical approach tailored for the analysis of genomic data based on the false discovery rate (FDR) expressed as a *q*-value [38]. For example, a 5% FDR, *q*≤0.05, indicates that 5% of the results judged to be significant could represent false positive signals. In addition, to improve the functional significance of our hits, we only considered absolute PSI value differences, |ΔΨ|, that were superior to 5 percentage points.

**Figure 1:**
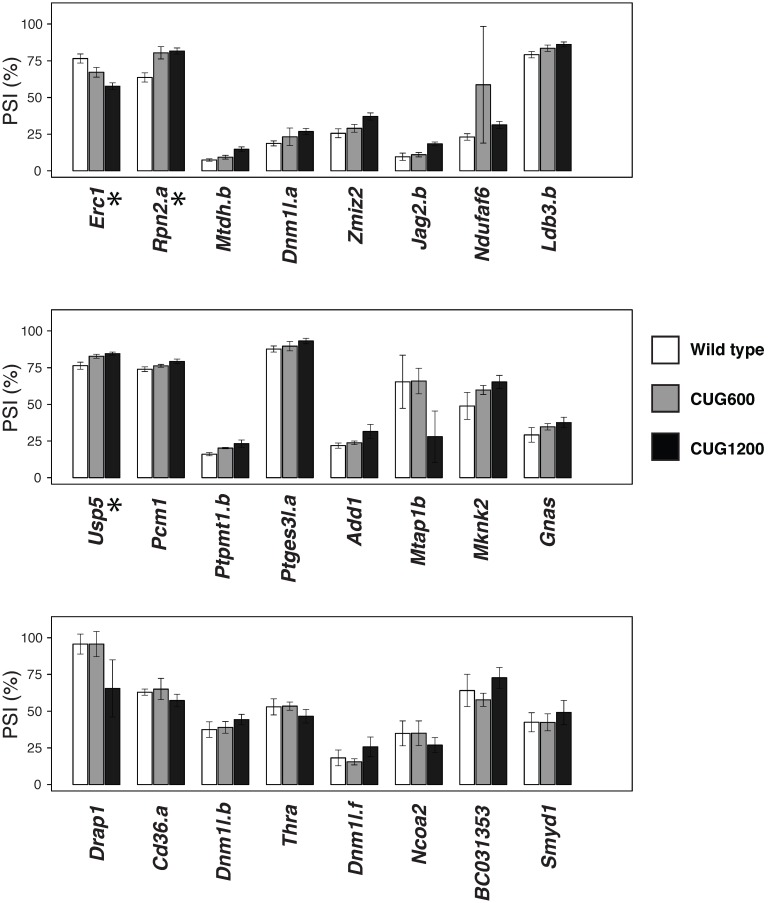
Splicing defects in a mouse strain expressing CUG repeats. Total RNA from muscle tissues of transgenic C57BL6 mice expressing 600 and 1200 CUG-repeats were screened for alternative splicing defects. We interrogated 172 ASEs in genes reported to be susceptible to changes in HSA^LR^ and MBNL knockout mice [14]. Using a false discovery rate threshold (*q-*value) of 0.05 and |ΔΨ| greater than 5 percentage points, we identified 24 ASEs in CUG1200 (black bars) that are significantly different from WT (white bars). Changes that were also significant in CUG600 (grey bars) are indicated with an asterisk. Results are presented in histograms by order of significance based on *q*-values.

Although each tissue for each mouse was analyzed individually, the results of the two tissues for each mouse were pooled in the final analysis because very few differences were noted between tissues (see below). Based on the above criteria, a total of 24 ASEs were identified as significantly misregulated in CUG1200 mice (**Fig. 1** **, Table S1**). From the set of 58 directly designed ASEs, 9 were in agreement with the misregulated events in the HSA^LR^ mouse, and 4 shifted in the opposite direction [14]. The remaining 11 misregulated ASEs were identified from the 114 indirectly designed ASEs, i.e. occurring in the same genes and overlapping regions reported in the HSR^LA^ study (**Table S1**). Three of the misregulated ASEs (*Erc1, Rpn2.a* and *Usp5*) were also significantly altered in CUG600 mice (marked with a * in **Fig. 1**). Other ASEs in the CUG600 samples had mean Ψ values consistent with a slight progression in the direction of the shift occurring in CUG1200 (e.g. *Mknk2, Mtdh, Dnm1l.a, Ldb3b, Gnas, Ptpmt1.b* and *Zmiz2*). The splicing profiles between tibialis anterior and gastrocnemius were very similar except for 5 ASEs (*Mtdh.a, Opa1, Picalm.b, Spag9* and *Smyd1*) (**Table S1**). Of these, only *Smyd1* was mis-spliced in CUG600/1200 hits. Of the ensemble of CUG1200 hits, *Usp5* and *Drap1* shifted in the ΔMBNL, but not in the HSA^LR^ mice based on microarray analysis [14]. The most dramatic shifts occurred in *Mtap1* and *Drap1*, with a greater than 20 point drop in exon inclusion in CUG1200 mice (**Fig. 1**). DRAP1 is a transcription factor that can interact with FEZ1, a protein involved in axon growth in nematodes [39]. *Mtap1*, also known as *MAP1B*, encodes the microtubule associated protein 1b involved in the cross-bridging between microtubules and other cytoskeletal elements in neurons, and can interact with actin and signaling proteins [40]. Interestingly, CTG repeats disturb the expression and subcellular distribution of the related and interacting partner MAP1A in a neuronal cellular model [41], suggesting that the change in *MAP1B* splice variants may contribute to this redistribution.

### Mis-splicing events in human embryonic DM1 myoblast primary cultures

More than 50 splicing alterations have been identified in skeletal and cardiac muscle of adult humans suffering from DM1 [8], [14], [42]. Overall, the defects indicate an incapacity to engage in a postnatal splicing transition [2], [22]. To address the extent of defective alternative splicing regulation in the developing muscle of DM1 embryos, we produced human DM1 myoblastic primary cultures from embryonic muscle tissues carrying 750, 1200 and 3500 CUG repeats (ST-750, ST-1200 and ST-3500, respectively). Three cultures were produced from each original sample. Normal embryonic and adult muscle cultures were also produced and used as controls.

**Figure 2:**
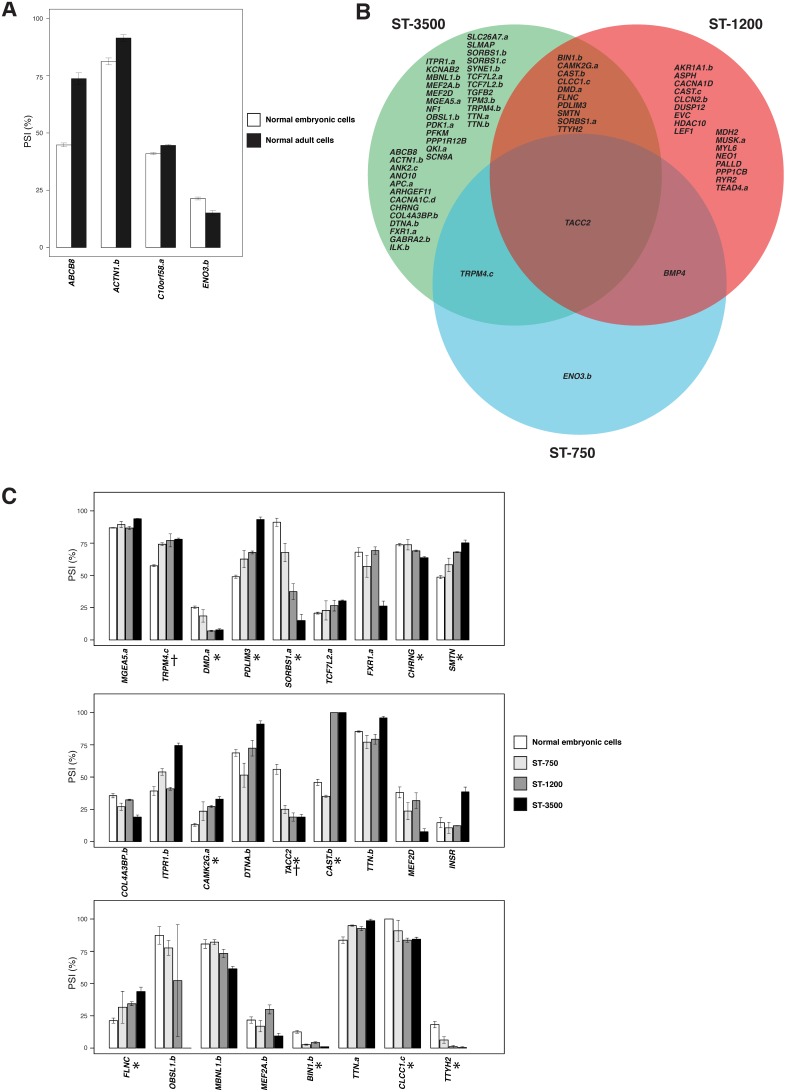
Differences in alternative splicing events (ASEs) when normal adult muscle cell lines are compared to normal embryonic muscle cell lines. (**A–C**) **A.** Histograms representing the four ASEs (*ABCB8*, *C10orf58*, *ACTN1*, *ENO3*) that are differentially spliced when normal embryonic cell lines (white bars) are compared to normal adult cell lines (black bars). **B.** Venn diagram representing hits when the three embryonic cell line categories (ST-750, ST-1200, and ST-3500) were compared to normal fetal cell lines. **C.** Histograms representing Ψ values in the normal fetal (white bars), ST-750 (light grey bars), ST-1200 (dark grey bars) and ST-3500 cells (black bars). Only the top 27 of the 50 splicing alterations seen in ST-3500 (*q* ≤ 0.05) are shown which also include all ST-750 (†) and ST-1200 (*) hits (respective *q*≤0.05) relative to normal fetal cells.

A detection screen using pooled RNA from normal and DM1 cultures was first carried out to identify splice variants for which the less abundant form represented at least 10% of the sum of both variants. Out of 2034 known human ASEs in muscle-relevant genes, we identified 487 such ASEs. We used this set first to compare myoblast cultures from normal adult and normal embryonic muscles (3 cultures each). A T-test on these two sample sets revealed fifty statistically significant ASEs, *p*<0.05 (**Table S2, DM1 fetal cells tab, column W**). Of these, the top four events that differentiate normal embryonic from normal adult muscle primary myoblastic cultures were *ABCB8*, *C10orf58*, *ACTN1* and *ENO3* (**Fig. 2a**).

Next, comparing splicing in DM1 and normal embryonic primary cultures identified 50 ASEs that were differently spliced between the embryonic ST-3500 and the normal embryonic cultures (*q* <0.05 and |ΔΨ| >5 percentage points) (**Table S2, DM1 fetal cells tab, columns B and T,** **Fig. 2b and 2c**). For 6 of these, *PDLIM3, SMTN, TACC2, BIN1.b, PPP1R12B* and *SORBS1.c*, the defects suggested an exacerbated embryonic splicing profile. The ST-1200 cultures yielded 29 hits, 11 of them seen in ST-3500 and displaying a good correlation between the amplitude of the splicing alterations and the number of repeats in the expansion (**Fig. 2b and 2c** **,** **Table S2, DM1 fetal cells tab, column R**). The ST-750 myoblasts only produced 4 hits, three of them occurring either in the ST-1200, ST-3500 or both (*TACC2*) (**Fig. 2b and 2c** **, Table S2, DM1 fetal cells tab, column P**). While all the differences noted were statistically significant, a subset in each category (e.g., the ASEs affected in ST-750 but not in ST-3500) may reflect individual-specific splicing differences or differences in the status of embryonic muscle differentiation at the time of collection. Thus, because some of the splicing differences may reflect genetic differences between the individuals, splicing differences that are common to ST-3500 and ST-1200 are likely to be most relevant to DM1.

On the other hand, we observed more alterations in the ST-3500 samples, which is what would be expected. Of the 50 muscle-relevant, statistically significant alternative splicing aberrations in the ST-3500 myoblast cultures, one mis-splicing event in *BIN1* (exon 11) associates with T tubule alterations and muscle weakness in myotonic dystrophy [12] (**Fig. 2c**). Exon 11 skipping in the insulin receptor (*INSR*) is one of the first alternative splicing events described to be aberrant in DM1 patients [11]. Although *INSR* splicing was aberrant in the embryonic ST-3500 cell lines (**Fig. 2c**), it occurred in the direction opposite to adult DM1 (i.e., more inclusion in fetal DM1 and more skipping in adult DM1).

Since the 487 ASEs that were screened were selected for their relevance to muscle function, splicing differences may affect muscle function. Functional gene ontology annotation using GOrilla [43] revealed a greater than 3-fold enrichment for processes related to cytoskeleton and actin-linked cytoskeleton function (*p* values of 3.4×10^−4^ and 9.2×10^−4^, respectively), as well as a 2.5-fold enrichment for cytoskeleton, as a cellular compartment. The cytoskeleton-related genes in these lists were *SYNE1*, *APC*, *MEF2A*, *OBSL1*, *TTN*, *PDLIM3*, *NF1*, *TACC2*, *ACTN1*, *SORBS1*, *DMD*, *SLMAP*, *TPM3*, *ANK2*, *BIN1* and *FLNC*. The enrichment is even stronger if we include *MEF2D* [44] and the cytoskeleton-associated protein *SMTN* (smoothelin)[45]. We also noted splicing alterations in several channel genes including the transient receptor potential cation channel *TRPM4*, the sodium channel *SCN9A*, the chloride channel *CLCC1*, the voltage-gated potassium channel K_v_4.3 gene *KCND3*, and the voltage-dependent calcium channel *CACNA1C*. In the case of *CACNA1C*, the variant made in DM1 cells changes the kinetics and voltage-dependence of inactivation as well as recovery from inactivation [46].

### Altered splicing in human adult DM1 tissues

**Figure 3:**
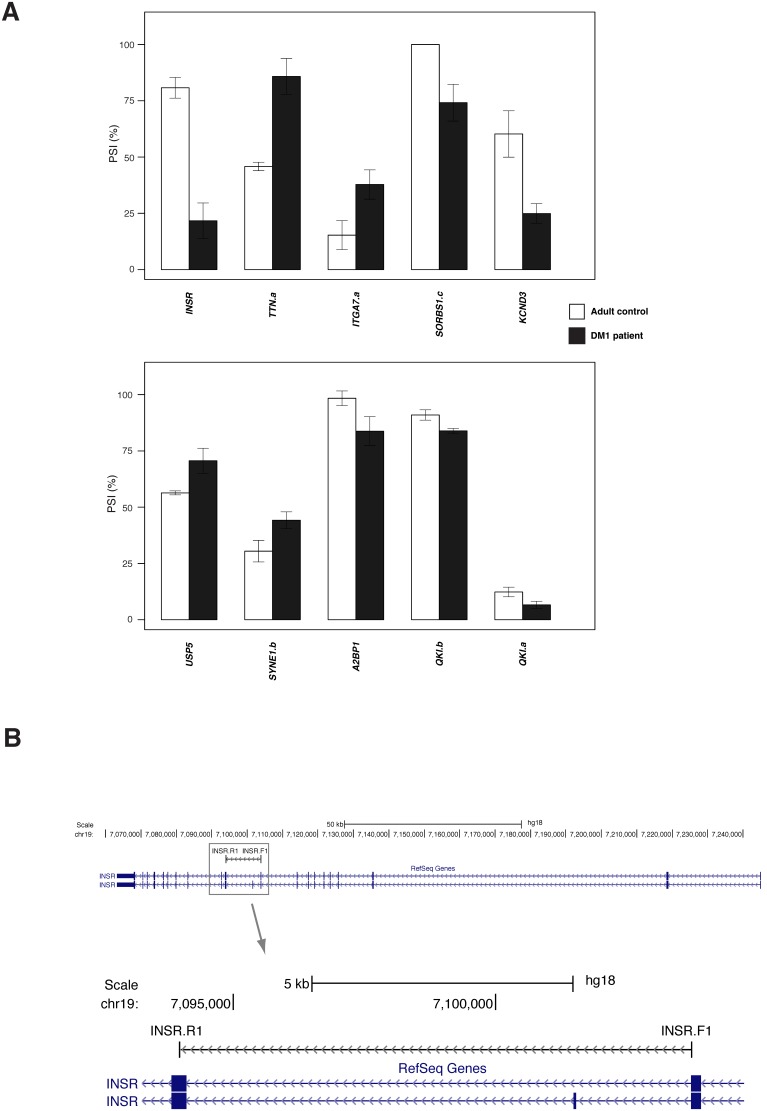
Splicing defects in DM1 patient tissues. (**A–B**) **A.** Ψ values for ten misspliced ASEs are represented as histograms for 4 adult controls (white bars) and 5 DM1 patients (black bars). Error bars represent standard deviations for each ASE. Hits were defined as changes displaying *q* values <0.05 and |ΔΨ| >5%. **B.** Sample UCSC Genome Browser (http://genome.ucsc.edu) adaptation showing the chromosome 19 region harboring human insulin receptor, INSR. Top image shows reported full-length RefSeq transcripts, the targeted ASE is boxed and shown in detail in the bottom image. The positions and names of the primers used for mRNA amplification by RT-PCR are shown above the transcripts. Links to transcript maps and primer positions for all human ASEs studied here can be found at http://palace.lgfus.ca/data/related/2073/odgene_/.

Next, we asked whether any of the splicing alterations identified in DM1 fetal cultures also occurred in adult DM1 tissues. Using five adult DM1 and four normal tissues, we selected 163 ASEs based on *q* values (less than 0.1) for any of the previous 5 comparisons: fetal vs. ST-750, fetal vs. ST-1200, fetal vs. ST-3500, fetal vs. combined ST-750, ST-1200 and ST-3500 and fetal vs. adult. Following the analysis on adult tissues, we identified 10 events altered in DM1 with |ΔΨ| and *q* values of 5 percentage points and <0.05, respectively (**Fig. 3** **, Table S2, DM1 tissues tab**). Of these, 6 were aberrantly spliced in the fetal DM1 cultures (*INSR, TTN.a, SORBS1.c, KCND3, SYNE1.b* and *QK1.a*). The exon inclusion event in a titin family member (*TTN.a*) occurs at the 3′ end of the coding region [47], as similarly noted in all fetal DM1 cell lines. For *INSR*, *SYNE1.b* and *QK1.a,* splicing in DM1 adult tissues occurred in the direction opposite to the shift seen in embryonic DM1 cultures. Although *ITGA7*, *A2BP1, USP5* and *QK1.b* were slightly below our cut-off in the fetal DM1 cultures, *A2BP1* and *USP5* were hits in DM1 mouse models [14]. *ITGA7* encodes an integrin that plays a role in acetylcholine receptor clustering when neuromuscular junctions are formed [48]. We also considered the next 20 strongest mis-spliced events in adult DM1 tissues (**Table S2, DM1 tissues tab, blue coloring in column E**). Seven hits in this category were also seen as fetal DM1 hits (*ANK2.c, TRPM4.b, CLCC1.c, SORBS1.a, TRPM4.c, MGEA5.a* and *CAST*; the three underlined events shift in the opposite direction of fetal DM1). Overall, 21% of the 30 adult DM1 hits were also hits in the embryonic DM1 cell cultures. Events that shifted in the same direction may reflect a DM1 fetal splicing profile in the regenerating fibers of adult DM1 tissues [49]. Thus, although some splicing defects are common in embryonic and adult muscle, others reflect embryonic- and adult-specific splicing alterations.

### MBNL1 and RBFOX1 co-regulate a subset of events altered in DM1

**Figure 4:**
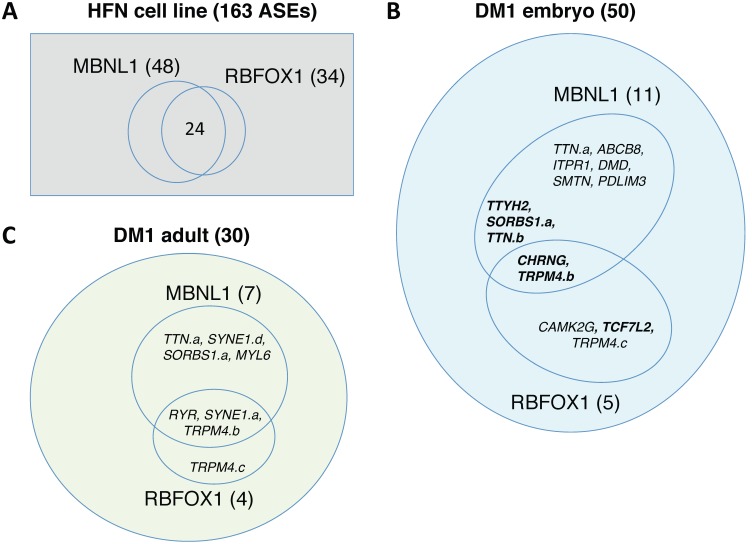
Role of MBNL1 and RBFOX1 in splicing regulation. (**A–C**) **A.** Venn diagram representing the overlap of hits obtained by knocking down MBNL1 and RBFOX1 in the HFN embryonic muscle cell line. In panels **B** and **C**, Venn diagrams are presented to illustrate events coregulated by MBNL1 and RBFOX1 that are mis-spliced in embryonic DM1 lines or and DM1 adult samples. The number and identity of the ASEs in each category are indicated. Gene names in bold indicate that the splicing shift for those ASEs occur in the reverse direction to the DM1 mis-splice.

To assess the contribution of the regulatory splicing factor MBNL1 to the human DM1 splicing alterations, we knocked down MBNL1 by RNA interference in a normal muscle embryonic cell culture (HFN) and performed the splicing analysis in triplicate. The depletions were confirmed by RT-qPCR (**Table S2, qPCR tab**). We interrogated the 163 ASEs used in the previous section, and identified 48 that were regulated by MBNL1 (**Table S2, MBNL1 RBFOX1 knockdown tab**, **Fig. 4a**). Eleven of the MBNL1-responsive events were mis-spliced in the DM1 embryonic cultures, five of them shifting in the direction opposite to DM1 (**Fig. 4b**). The MBNL1 depletion affected only one of our 10 strongest adult DM1 mis-splicing events, and 6 of the 20 successive alterations (Blue colored hits in **Table S2, DM1 tissues tab, column E, and** **Fig. 4c**). These 7 shifts occurred in the same direction in ΔMBNL1 and DM1 tissues.

Alternative splicing of *A2BP1* (a.k.a. *RBFOX1*) was affected in DM1 adult tissues (|ΔΨ| = 15 percentage points, *q* = 0.04) (**Fig. 3a**). In the DM1 fetal culture, it yielded a |ΔΨ| of 10 points, but with a *q* value above our threshold (0.08). Although not a hit with the CUG1200 mice, the *A2BP1* gene is mis-spliced in the ΔMBNL1 mice [14]. In all cases, a skipping event produces a variant that lacks a portion of the RNA recognition motif (RRM) domain involved in recognizing the regulatory sequence (^U^/_A_)GCAUG [50]–[54]. RBFOX1 is specifically expressed in neurons, heart and muscle [52], [55]. RBFOX proteins are important regulators of muscle function in zebrafish where their depletion affects myofiber development [56]. RBFOX1 regulates the alternative splicing of many critical transcripts essential for neuronal excitation and synaptic transmission [57]. We asked if RBFOX1 regulates the splicing of muscle-relevant genes. Performing the depletion of RBFOX1 in the normal HFN culture (in triplicate) revealed 34 splicing events that were sensitive to a decrease in RBFOX1 (50% drop based on qRT-PCR) (**Fig. 4** **; Table S2 qPCR tab**). Although it is unclear if the 10–15% increase in the RBFOX1ΔRRM variant occurring in DM1 tissues would be sufficient to alter the splicing regulation of target ASEs, some impact may be expected because this variant displays dominant negative activity [54]. Consistent with this view, 5 of the 34 RBFOX1-sensitive events were misregulated in embryonic DM1 cultures (*CAMK2G, TCF7L2, TRPM4.b, TRMP4.c and CHRNG*), and 4 were affected in adult DM1 (*RYR, SYNE1.a, TRMP4.b* and *TRMP4.c*). Strikingly, half of the 48 events regulated by MBNL1 were also regulated by RBFOX1 (**Fig. 4a**). Moreover, 5 of 9 RBFOX1 hits that were mis-spliced in DM1 were also regulated by MBNL1 (**Fig. 4b and 4c**). We confirmed this overlapping regulation by repeating the RBFOX and MBNL1 depletions in a different HFN cell culture. Even with partial depletions, as judged by western analysis (**Fig. S1a**), 11 of 13 events that were regulated by MBNL1 were sensitive to the depletion of RBFOX (**Fig. S1b**).

## Discussion

Mouse model systems have been developed to reproduce the molecular and physiopathological deficiencies found in DM1 patients. Although none of the current models displays the full repertoire of physiological deficiencies observed in DM1 patients, they nevertheless offer the possibility of associating specific phenotypes with molecular alterations, and identifying discerning features that might explain shared or specific defects. The Ares group previously identified a collection of splicing alterations in MBNL1 knockout mice and HSA^LR^ mice that express CUG-repeats [14]. Of these, 4 of the 6 human orthologous ASEs were affected in three DM1 individuals. Of the 11 orthologous ASEs derived from hits reported in the Ares study, we found 4 (*USP5, TACC2, CAMK2* and *A2BP1*) that were affected in 9 embryonic myoblast cell cultures and 5 adult patient muscle tissues. Using a stringent set of criteria for revealing splicing alterations in muscle tissues (splicing shifts greater than 5 percentage points and *q*≤0.05), only 5 of the 33 original validated mouse hits uncovered in the Ares study were similarly mis-spliced in our CUG1200 mice. Although the smaller set of molecular alterations that we identified may be attributable to differences in the detection methods, different levels of transgene expression likely contributed to the discrepancies. Indeed, the CUG repeats of the CUG1200 mice are imbedded in a transgenic *DMPK* gene whose expression level in muscle is five times lower than the murine endogenous gene [30], whereas the transcript carrying the 250 CUG repeats of the HSA^LR^ mice is expressed at a much higher level [24]. Consistent with this view, CUG1200 mice have a phenotype that is milder than the MBNL1 knockout and HSA^LR^ mice [30]. It is also possible that alternative splicing events in C57BL/6 mice are in general less sensitive to expression of CUG repeats. *SCNM1* is a disease modifying allele that affects splicing, and that is most severely affected in C57BL/6 [58], [59]. Such strain-specific differences may produce overlapping but globally distinct splicing signatures that may contribute to differences in the expression of the DM1 phenotype in FVB/n and C57BL/6 mice. Because no animal model reproduces the full pathophysiological manifestations of the disease, it is therefore of utmost importance to use human samples to validate data obtained from mouse models with the goal of uncovering molecular alterations that associate with core aspects of the pathophysiology.

We identified 50 mis-splicing events in the fetal DM1 myoblast cell cultures, including events known to be mis-spliced in adult tissues, such as *BIN1, INSR, CLCC1, TTN.a* and *TTN.b* [42]. We also confirmed a known misregulated splicing event at the 3′ end of the coding region of *DMD* (dystrophin), a gene in which mutations cause Duchenne and Becker muscular dystrophies. Overall, half of the altered events in embryonic DM1 cells occurred in cytoskeleton (21 ASEs) and channel (5 ASEs) genes. Among the channel genes, the voltage-gated potassium channel K_v_4.3 gene *KCND3* produced more of the skipped product both in embryonic and adult DM1 cells. KCND3 is involved in neuronal excitability and is a target of the splicing factor RBFOX1, since inclusion of the 57 nt exon is increased in the brain of *Rbfox1*−/− knockout mice [57]. We observed that human *KCND3* splicing remained unchanged when RBFOX1 was knocked down, possibly because the intron upstream of the alternative exon in human *KCND3* lacks the RBFOX binding element and putative silencer found in the mouse gene at this position [57].

By profiling splicing in adult DM1 tissues, we noted two known aberrant mis-splicing events (*INSR* and *TTN.a*), but also identified several new alterations. In addition to *KCND3*, *SYNE1* splicing was also altered both in embryonic and adult DM1 tissues. SYNE1 (also known as nesprin) is a spectrin-repeat protein that forms a network that links various subcellular structures throughout the muscle sarcomere to the actin cytoskeleton. *SYNE1* has been implicated in Emery-Dreifuss muscular dystrophy (muscle wasting and weakness) [60]. The alternative segment of SYNE1 does not affect the structure of known protein functional domains; thus the functional impact of the variant lacking the alternative exon remains unclear. *QKI.a* splicing was altered in DM1 patients (more inclusion). QKI is an RNA binding protein involved in neuronal function (myelination), blood vessel formation, smooth muscle formation and heart development. In zebrafish, the loss of *QkA* affects fast muscle fiber maturation as well as Hh-induced muscle derivative specification and/or morphogenesis [61], [62]. QKI was recently implicated as a global regulator of splicing during vertebrate muscle development [63].

### Regulatory interactions of MBNL1 and RBFOX1

We sought to gain insights into the regulatory pathways that yield splicing alterations in fetal and adult human DM1 muscle tissues. The impact of depleting MBNL1 was evaluated because the sequestration of MBNL1 by CUG repeats has been associated with many splicing defects. The depletion of MBNL1 in the embryonic myoblast culture (HFN) revealed 48 alternative splicing events sensitive to MBNL1 levels (**Table S2, MBNL1 RBFOX1 knockdown tab, and** **Fig. 4a**). Six of these events occurred in the same direction as in the fetal DM1 lines, and five occurred in the reverse direction (**Fig. 4b**).

We also tested the impact of RBFOX1 because it is mis-spliced in adult DM1 tissues and in ΔMBNL1 mice [14] to produce a defective regulator lacking a complete RNA binding motif [54]. A recent study indicated that the knockdown of *Rbfox1* inhibits muscle differentiation, and that RBFOX1 expression was altered in a mouse model of facioscapulohumeral muscular dystrophy [64]. We identified 34 RBFOX1 targets in muscle-relevant genes (**Fig. 4a**), seven of which displayed altered splicing in embryonic and/or adult DM1 samples (**Fig. 4b and 4c**). Notably, while the individual depletion of MBNL1 and RBFOX1 respectively affected the splicing of 29% and 20% of the 163 events tested, half of the 48 events controlled by MBNL1 were co-regulated by RBFOX1. In addition to ion channel proteins and components of the cytoskeleton, the list (**Table 1**) includes acetylcholine receptors, which in conjunction with integrins, are critical to form neuromuscular junctions [48]. The MBNL1/RBFOX1 co-regulated genes also include four that were mis-spliced in DM1 tissues (**Fig. 4b and 4c**). Knocking down MBNL1 and RBFOX2 in a cancer cell line revealed a similar convergence of regulation for muscle-relevant genes since 50% of these genes were co-regulated by MBNL1 and RBFOX1 (http://palace.lgfus.ca/data/related/2075). In contrast, only 6% of co-regulation was observed on a different set of 47 genes implicated in cancer [65]. While MBNL1 and RBFOX1 have been individually implicated in modulating splicing decisions during muscle and heart development [2], [66], our results suggest that MBNL1 and RBFOX proteins converge to regulate the splicing of a common subset of genes involved in muscle function. Recent work suggests that MBNL1 and RBFOX2 also cooperate to implement a splicing program associated with the differentiation of human stem cells [67]. It is therefore intriguing to postulate that a partial loss of MBNL1 and RBFOX1 activity in both fetal and adult DM1 tissues may compromise a critical splicing program associated with muscle differentiation. Given that muscle differentiation occurs in the damaged skeletal muscle of DM1 mice [49], mis-splicing events in muscle-relevant genes caused by defective MBNL1 and RBFOX1 activity may compromise tissue regeneration. Lastly, it is interesting to consider the microtubule-associated protein Tau (MAPT/Tau) whose splicing is misregulated in DM1 brains [68], [69], a defect that may contribute to neuropsychological manifestations. MBNL proteins regulate the splicing of *tau* alternative exon 2 [70], and we find that ectopic expression of RBFOX1 can partially repress the splicing aberration of *tau* exon 2 induced in T98 glioblastoma cells by transfecting the CUG repeat expression vector DT960 (**Fig. S2**). Co-regulation of splicing by MBNL and RBFOX proteins may therefore extend to neuronal tissues, and their deficient activity in DM1 brains may lead to aberrant splicing of genes such as *tau/MAPT* that may then contribute to cognitive abnormalities.

**Table 1:** Alternative splicing events co-regulated by MBNL1 and RBFOX1.

Gene	Description	Function	Association with diseases
**Neuromuscular Organogenesis**			
**CHRNG**	acetylcholin reeceptor γ-subunit	neuromuscular junction	
CHRNA5	acetylcholine receptor	neuromuscular junction	
ACHE	acetylcholinesterase	neuromuscular junction	
ILK	integrin-linked kinase	neuromuscular junction	
EVC	transmembrane	muscle development	Ellis-van Creveld syndrome
**Channels**			
**RYR1** (2)	calcium release channel	connects sarcoplasmic reticulum with tubules	minicore myopathy
CLCN2	chloride channel		epilepsy
CACNA1C	calcium channel		
KCNK2	potassium channel		
**TRPM4**	calcium transport		
CATSPER3	calcium channel		
**Others**			
JPH4	junctophilin	plasma membrane and sarcoplasmic reticulum	
NEB	nebulin	cytoskeletal matrix in sarcomeres	nemaline myopathy
BMP4	TGF-β superfamily	bone morphogenesis	
CAPN3	calpain 3	putative protease that binds to titin	limb-girdle muscular dystrophies type 2A
TAZ	membrane associated		cardiomyopathy
**SYNE1**	spectrin-repeat containing protein	nuclear membrane	Emery–Dreifuss muscular dystrophy
MYLK	myosin light chain kinase		
PHKB	phosphorylase kinase		
TBC1D1		cell differentiation	
SORBS2	sorbin, tyrosine kinase	assembly of signalling complex in stress fibers	
MDH2	malate dehydrogenase		
ITPR1	intracellular receptor for inositol 1,4,5-trisphosphate		spinocerebellar ataxia

Genes in bold are mis-spliced in DM1 tissues.

Overall, our analysis has unveiled a splicing regulatory network where MBNL1 and RBFOX1 are co-regulating a group of events that may be relevant to muscle function and development. Since RBFOX1 most often imposes regulation in the same direction as MBNL1, the aberrant splicing of RBFOX1 in adult DM1 tissues may therefore amplify the mis-splicing of ASEs already affected by the MBNL1 deficiency.

## Supporting Information

Figure S1 **ASEs that are co-regulated by MBNL1 and RBFOX1/RBFOX2. A.** Immunoblot analysis following the knockdown of RBFOX1/RBFOX2 and MBNL1 in a HFN cell culture. A control siRNA (si-C) was also used, and housekeeping proteins (GAPDH or α-tubulin) were tested as loading controls. **B.** Histograms showing splicing changes (ΔΨ) observed for ASEs following MBNL1 (black bars) or RBFOX1/2 (white bars) knockdown, relative to si-C-treated HFN cells.
(PDF)

Figure S2 **RBFOX1 represses the impact of CUG-repeats on** ***tau*** **splicing in human glioblastoma T98G cells.** T98G cells were transfected with expression vectors for RBFOX1, CUG-repeats (DT960) or both. Agarose gel of RT-PCR reactions designed to amplify *tau* splicing products are shown on top, and histograms depict exon 2 exclusion level in percentage with standard deviations. * = *p*<0.05; ** = *p*<0.01. In non-DM1 mimicking conditions, RBFOX1 did not significantly modify the splicing of *tau* exon 2. DT960 increased *tau* exon 2 exclusion, and this effect was partially prevented by co-expressing RBFOX1.
(PDF)

Table S1 **List of ASEs screened in mice.** The table lists all ASEs that were tested for splicing in mouse tissues. For each ASE, the gene name and primer pair names (Columns A and B) and a summary description of the type and size of each ASE (Column C) is given. Column D provides a comparison of PSI shift direction between our observations and those reported by Du et al. [14]. *p*-values, *q*-values and |ΔΨ| are also provided for the CUG600 and CUG1200 mice relative to wild type (WT), as well as for the gastrocnemius and tibialis anterior comparison (Columns E-M). Tests for threshold levels of *p*, *q* and |ΔΨ| are reported in Columns N-AB. Average PSI values and standard deviations are presented in Columns AC-AH for all tissue groups screened. Column AI compares hits obtained with data from Du et al. [14]. The portion in yellow corresponds to misregulated events in CUG1200 with *q*-values inferior to 0.05 and |ΔΨ| superior to 5 percentage points. When more than 1 ASEs originated from one gene, ASEs were categorized as xxx.a, xxx.b, etc.
(XLSX)

Table S2 **List of ASEs screened in DM1 embryonic cell lines, patient tissues and knockdown assays.** The file includes three sheets that individually list the ASEs screened in each experiment. **DM1 Fetal Cells tab:** List of the 487 human ASEs analyzed. Column A: Gene Name, where more than one ASE was targeted the suffix.a,.b, … is used. Column B: |ΔΨ| values for ST-3500 (Ψ values: Column M, standard deviation: Column N) minus Normal Fetal cells (Ψ values: Column E, standard deviation: Column F). Columns C and D: hit in cells (this sheet) and DM1 tissues (DM1 Tissues sheet) respectively, refer to text for selection criteria. Fifty cell hits shown in red, tissue hits shown in yellow and blue (see legend for DM1 Tissues sheet, below). Columns G-L: Ψ values and standard deviations for Normal Adult, ST-750 and ST-1200 cells. Columns O-T: Calculated *p* and *q* values (see text) for Normal Fetal cells (N_F) compared to ST-750, ST-1200 and ST-3500 cells. Columns U and V: *p* and *q* values for Normal fetal cells compared to all three DM cell lines combined. Columns W and X: *p* and *q* values for Normal fetal cells compared to Normal Adult (N_A) cells. **DM1 Tissues tab:** List of the 163 human ASEs analyzed. Column A: Column A: Gene Name, where more than one ASE was targeted the suffix.a,.b, … is used. Columns B-D: ASE type and expected amplicon sizes in base pairs (bp) following PCR amplification of region flanking ASE. Column E and F: hit in tissues (this sheet) and cells (DM1 Fetal Cells sheet) respectively, refer to text for selection criteria. Top ten tissue hits are shown in yellow, and following 20 hits shown blue. Columns G-I: |ΔΨ|, *p* and *q* values for DM1 tissues versus normal controls. Columns J-R: Ψ values for individual DM1 and control (CTL) tissues. Column S-V: PCR primer names and sequences. **MBNL1 RBFOX1 Knockdown tab:** List of the 163 human ASEs analyzed (identical to DM1 Tissues). Column A: Gene Name, where more than one ASE was targeted the suffix.a,.b, … is used. Columns B and C: Hit in tissues (DM1 Tissues sheet) and cells (DM1 Fetal Cells sheet) respectively, refer to text for selection criteria. Top ten tissue hits are shown in yellow, and following 20 hits shown blue, 50 cell hits shown in red. Columns E-K: |ΔΨ| MBNL1 or RBFOX1 knockdown minus control in HFN and fibroblasts, see text for hit criteria. Columns L-S: ΔΨ data for untreated HFN, mock transfected HFN, MBNL1 and RBFOX1 knockdown HFN cells and untreated and knockdown fibroblast cells. Column T: Presence and position relative to splice site of RBFOX1 binding motif. Negative number is nucleotide position upstream of 3′ splice site, positive number is position relative to 5′ splice site of alternative exon. Column U and V: Presence, occurrence and position relative to splice sites of UGC repeats (e.g. 2×1 refers to two repeats occurring 1 time). Positions upstream (negative numbers) and downstream (positive numbers) relative to 3′ and 5′ splice sites, respectively. **qPCR tab:** mRNA expression levels following RNA interference knockdown of MBNL1 and RBFOX1. Relative expression levels (RE) and technical error of triplicate qPCR reactions (dT) shown 48h post transfection for mock transfected HFN cells (HFN CTL-), and knockdowns (HFN siMBNL, and HFN siRBFOX1). Reference gene primer sequences are shown. RE and dT calculations performed using the qBASE package [34].
(XLSX)

Text S1 **Supplementary material and methods.**
(DOCX)

## References

[1] Taneja KL, McCurrach M, Schalling M, Housman D, Singer RH (1995) Foci of trinucleotide repeat transcripts in nuclei of myotonic dystrophy cells and tissues. J Cell Biol 128: 995–1002. 7896884 10.1083/jcb.128.6.995 PMC2120416

[2] Kalsotra A, Xiao X, Ward AJ, Castle JC, Johnson JM, et al (2008) A postnatal switch of CELF and MBNL proteins reprograms alternative splicing in the developing heart. Proc Natl Acad Sci U S A 105: 20333–20338. 19075228 10.1073/pnas.0809045105 PMC2629332

[3] Timchenko NA, Cai ZJ, Welm AL, Reddy S, Ashizawa T, et al (2001) RNA CUG repeats sequester CUGBP1 and alter protein levels and activity of CUGBP1. J Biol Chem 276: 7820–7826. 11124939 10.1074/jbc.M005960200

[4] Paul S, Dansithong W, Kim D, Rossi J, Webster NJ, et al (2006) Interaction of muscleblind, CUG-BP1 and hnRNP H proteins in DM1-associated aberrant IR splicing. EMBO J 25: 4271–4283. 16946708 10.1038/sj.emboj.7601296 PMC1570429

[5] Mankodi A, Lin X, Blaxall BC, Swanson MS, Thornton CA (2005) Nuclear RNA foci in the heart in myotonic dystrophy. Circ Res 97: 1152–1155. 16254211 10.1161/01.RES.0000193598.89753.e3

[6] Kuyumcu-Martinez NM, Wang GS, Cooper TA (2007) Increased steady-state levels of CUGBP1 in myotonic dystrophy 1 are due to PKC-mediated hyperphosphorylation. Mol Cell 28: 68–78. 17936705 10.1016/j.molcel.2007.07.027 PMC2083558

[7] Kuyumcu-Martinez NM, Cooper TA (2006) Misregulation of alternative splicing causes pathogenesis in myotonic dystrophy. Prog Mol Subcell Biol 44: 133–159. 17076268 10.1007/978-3-540-34449-0_7 PMC4127983

[8] Gomes-Pereira M, Cooper TA, Gourdon G (2011) Myotonic dystrophy mouse models: towards rational therapy development. Trends Mol Med 17: 506–517. 21724467 10.1016/j.molmed.2011.05.004 PMC3881009

[9] Mankodi A, Takahashi MP, Jiang H, Beck CL, Bowers WJ, et al (2002) Expanded CUG repeats trigger aberrant splicing of ClC-1 chloride channel pre-mRNA and hyperexcitability of skeletal muscle in myotonic dystrophy. Mol Cell 10: 35–44. 12150905 10.1016/s1097-2765(02)00563-4

[10] Charlet BN, Savkur RS, Singh G, Philips AV, Grice EA, et al (2002) Loss of the muscle-specific chloride channel in type 1 myotonic dystrophy due to misregulated alternative splicing. Mol Cell 10: 45–53. 12150906 10.1016/s1097-2765(02)00572-5

[11] Savkur RS, Philips AV, Cooper TA (2001) Aberrant regulation of insulin receptor alternative splicing is associated with insulin resistance in myotonic dystrophy. Nat Genet 29: 40–47. 11528389 10.1038/ng704

[12] Fugier C, Klein AF, Hammer C, Vassilopoulos S, Ivarsson Y, et al (2011) Misregulated alternative splicing of BIN1 is associated with T tubule alterations and muscle weakness in myotonic dystrophy. Nat Med 17: 720–725. 21623381 10.1038/nm.2374

[13] Osborne RJ, Lin X, Welle S, Sobczak K, O'Rourke JR, et al (2009) Transcriptional and post-transcriptional impact of toxic RNA in myotonic dystrophy. Hum Mol Genet 18: 1471–1481. 19223393 10.1093/hmg/ddp058 PMC2664149

[14] Du H, Cline MS, Osborne RJ, Tuttle DL, Clark TA, et al (2010) Aberrant alternative splicing and extracellular matrix gene expression in mouse models of myotonic dystrophy. Nat Struct Mol Biol 17: 187–193. 20098426 10.1038/nsmb.1720 PMC2852634

[15] Wang ET, Cody NA, Jog S, Biancolella M, Wang TT, et al (2012) Transcriptome-wide regulation of pre-mRNA splicing and mRNA localization by muscleblind proteins. Cell 150: 710–724. 22901804 10.1016/j.cell.2012.06.041 PMC3428802

[16] Huichalaf C, Sakai K, Jin B, Jones K, Wang GL, et al (2010) Expansion of CUG RNA repeats causes stress and inhibition of translation in myotonic dystrophy 1 (DM1) cells. FASEB J 24: 3706–3719. 20479119 10.1096/fj.09-151159 PMC2996918

[17] Timchenko NA, Wang GL, Timchenko LT (2005) RNA CUG-binding protein 1 increases translation of 20-kDa isoform of CCAAT/enhancer-binding protein beta by interacting with the alpha and beta subunits of eukaryotic initiation translation factor 2. J Biol Chem 280: 20549–20557. 15788409 10.1074/jbc.M409563200

[18] Machuca-Tzili LE, Buxton S, Thorpe A, Timson CM, Wigmore P, et al (2011) Zebrafish deficient for Muscleblind-like 2 exhibit features of myotonic dystrophy. Dis Model Mech 4: 381–392. 21303839 10.1242/dmm.004150 PMC3097459

[19] Hao M, Akrami K, Wei K, De Diego C, Che N, et al (2008) Muscleblind-like 2 (Mbnl2) -deficient mice as a model for myotonic dystrophy. Dev Dyn 237: 403–410. 18213585 10.1002/dvdy.21428

[20] Kim DH, Langlois MA, Lee KB, Riggs AD, Puymirat J, et al (2005) HnRNP H inhibits nuclear export of mRNA containing expanded CUG repeats and a distal branch point sequence. Nucleic Acids Res 33: 3866–3874. 16027111 10.1093/nar/gki698 PMC1176012

[21] Charizanis K, Lee KY, Batra R, Goodwin M, Zhang C, et al (2012) Muscleblind-like 2-mediated alternative splicing in the developing brain and dysregulation in myotonic dystrophy. Neuron 75: 437–450. 22884328 10.1016/j.neuron.2012.05.029 PMC3418517

[22] Lin X, Miller JW, Mankodi A, Kanadia RN, Yuan Y, et al (2006) Failure of MBNL1-dependent post-natal splicing transitions in myotonic dystrophy. Hum Mol Genet 15: 2087–2097. 16717059 10.1093/hmg/ddl132

[23] Orengo JP, Chambon P, Metzger D, Mosier DR, Snipes GJ, et al (2008) Expanded CTG repeats within the DMPK 3′ UTR causes severe skeletal muscle wasting in an inducible mouse model for myotonic dystrophy. Proc Natl Acad Sci U S A 105: 2646–2651. 18272483 10.1073/pnas.0708519105 PMC2268190

[24] Mankodi A, Logigian E, Callahan L, McClain C, White R, et al (2000) Myotonic dystrophy in transgenic mice expressing an expanded CUG repeat. Science 289: 1769–1773. 10976074 10.1126/science.289.5485.1769

[25] Gomes-Pereira M, Foiry L, Nicole A, Huguet A, Junien C, et al (2007) CTG trinucleotide repeat “big jumps”: large expansions, small mice. PLoS Genet 3: e52. 17411343 10.1371/journal.pgen.0030052 PMC1847694

[26] Kanadia RN, Johnstone KA, Mankodi A, Lungu C, Thornton CA, et al (2003) A muscleblind knockout model for myotonic dystrophy. Science 302: 1978–1980. 14671308 10.1126/science.1088583

[27] Ward AJ, Rimer M, Killian JM, Dowling JJ, Cooper TA (2010) CUGBP1 overexpression in mouse skeletal muscle reproduces features of myotonic dystrophy type 1. Hum Mol Genet 19: 3614–3622. 20603324 10.1093/hmg/ddq277 PMC2928132

[28] Koshelev M, Sarma S, Price RE, Wehrens XH, Cooper TA (2010) Heart-specific overexpression of CUGBP1 reproduces functional and molecular abnormalities of myotonic dystrophy type 1. Hum Mol Genet 19: 1066–1075. 20051426 10.1093/hmg/ddp570 PMC2830830

[29] Seznec H, Agbulut O, Sergeant N, Savouret C, Ghestem A, et al (2001) Mice transgenic for the human myotonic dystrophy region with expanded CTG repeats display muscular and brain abnormalities. Hum Mol Genet 10: 2717–2726. 11726559 10.1093/hmg/10.23.2717

[30] Huguet A, Medja F, Nicole A, Vignaud A, Guiraud-Dogan C, et al (2012) Molecular, physiological, and motor performance defects in DMSXL mice carrying >1,000 CTG repeats from the human DM1 locus. PLoS Genet 8: e1003043. 23209425 10.1371/journal.pgen.1003043 PMC3510028

[31] Furling D, Coiffier L, Mouly V, Barbet JP, St Guily JL, et al (2001) Defective satellite cells in congenital myotonic dystrophy. Hum Mol Genet 10: 2079–2087. 11590125 10.1093/hmg/10.19.2079

[32] Beaulieu D, Thebault P, Pelletier R, Chapdelaine P, Tarnopolsky M, et al (2012) Abnormal prostaglandin E2 production blocks myogenic differentiation in myotonic dystrophy. Neurobiol Dis 45: 122–129. 21742035 10.1016/j.nbd.2011.06.014

[33] Venables JP, Koh CS, Froehlich U, Lapointe E, Couture S, et al (2008) Multiple and specific mRNA processing targets for the major human hnRNP proteins. Mol Cell Biol 28: 6033–6043. 18644864 10.1128/MCB.00726-08 PMC2547008

[34] Hellemans J, Mortier G, De Paepe A, Speleman F, Vandesompele J (2007) qBase relative quantification framework and software for management and automated analysis of real-time quantitative PCR data. Genome Biol 8: R19. 17291332 10.1186/gb-2007-8-2-r19 PMC1852402

[35] Klinck R, Bramard A, Inkel L, Dufresne-Martin G, Gervais-Bird J, et al (2008) Multiple alternative splicing markers for ovarian cancer. Cancer Res 68: 657–663. 18245464 10.1158/0008-5472.CAN-07-2580

[36] Venables JP, Klinck R, Bramard A, Inkel L, Dufresne-Martin G, et al (2008) Identification of alternative splicing markers for breast cancer. Cancer Res 68: 9525–9531. 19010929 10.1158/0008-5472.CAN-08-1769

[37] Venables JP, Klinck R, Koh C, Gervais-Bird J, Bramard A, et al (2009) Cancer-associated regulation of alternative splicing. Nat Struct Mol Biol 16: 670–676. 19448617 10.1038/nsmb.1608

[38] Storey JD, Tibshirani R (2003) Statistical significance for genomewide studies. Proc Natl Acad Sci U S A 100: 9440–9445. 12883005 10.1073/pnas.1530509100 PMC170937

[39] Assmann EM, Alborghetti MR, Camargo ME, Kobarg J (2006) FEZ1 dimerization and interaction with transcription regulatory proteins involves its coiled-coil region. J Biol Chem 281: 9869–9881. 16484223 10.1074/jbc.M513280200

[40] Halpain S, Dehmelt L (2006) The MAP1 family of microtubule-associated proteins. Genome Biol 7: 224. 16938900 10.1186/gb-2006-7-6-224 PMC1779536

[41] Velazquez-Bernardino P, Garcia-Sierra F, Hernandez-Hernandez O, Bermudez de Leon M, Gourdon G, et al (2011) Myotonic dystrophy type 1-associated CTG repeats disturb the expression and subcellular distribution of microtubule-associated proteins MAP1A, MAP2, and MAP6/STOP in PC12 cells. Mol Biol Rep 39: 415–424. 21567201 10.1007/s11033-011-0753-y

[42] Yamashita Y, Matsuura T, Shinmi J, Amakusa Y, Masuda A, et al (2012) Four parameters increase the sensitivity and specificity of the exon array analysis and disclose 25 novel aberrantly spliced exons in myotonic dystrophy. J Hum Genet 57: 368–374. 22513715 10.1038/jhg.2012.37

[43] Eden E, Navon R, Steinfeld I, Lipson D, Yakhini Z (2009) GOrilla: a tool for discovery and visualization of enriched GO terms in ranked gene lists. BMC Bioinformatics 10: 48. 19192299 10.1186/1471-2105-10-48 PMC2644678

[44] Molinari S, Relaix F, Lemonnier M, Kirschbaum B, Schafer B, et al (2004) A novel complex regulates cardiac actin gene expression through interaction of Emb, a class VI POU domain protein, MEF2D, and the histone transacetylase p300. Mol Cell Biol 24: 2944–2957. 15024082 10.1128/MCB.24.7.2944-2957.2004 PMC371105

[45] Rensen S, Merkx G, Doevendans P, Geurts Van Kessel A, van Eys G (2000) Structure and chromosome location of Smtn, the mouse smoothelin gene. Cytogenet Cell Genet 89: 225–229. 10965129 10.1159/000015619

[46] Tiwari S, Zhang Y, Heller J, Abernethy DR, Soldatov NM (2006) Atherosclerosis-related molecular alteration of the human CaV1.2 calcium channel alpha1C subunit. Proc Natl Acad Sci U S A 103: 17024–17029. 17071743 10.1073/pnas.0606539103 PMC1636572

[47] Kolmerer B, Olivieri N, Witt CC, Herrmann BG, Labeit S (1996) Genomic organization of M line titin and its tissue-specific expression in two distinct isoforms. J Mol Biol 256: 556–563. 8604138 10.1006/jmbi.1996.0108

[48] Burkin DJ, Gu M, Hodges BL, Campanelli JT, Kaufman SJ (1998) A functional role for specific spliced variants of the alpha7beta1 integrin in acetylcholine receptor clustering. J Cell Biol 143: 1067–1075. 9817762 10.1083/jcb.143.4.1067 PMC2132957

[49] Orengo JP, Ward AJ, Cooper TA (2011) Alternative splicing dysregulation secondary to skeletal muscle regeneration. Ann Neurol 69: 681–690. 21400563 10.1002/ana.22278 PMC3082633

[50] Nakahata S, Kawamoto S (2005) Tissue-dependent isoforms of mammalian Fox-1 homologs are associated with tissue-specific splicing activities. Nucleic Acids Res 33: 2078–2089. 15824060 10.1093/nar/gki338 PMC1075922

[51] Auweter SD, Fasan R, Reymond L, Underwood JG, Black DL, et al (2006) Molecular basis of RNA recognition by the human alternative splicing factor Fox-1. EMBO J 25: 163–173. 16362037 10.1038/sj.emboj.7600918 PMC1356361

[52] Underwood JG, Boutz PL, Dougherty JD, Stoilov P, Black DL (2005) Homologues of the Caenorhabditis elegans Fox-1 protein are neuronal splicing regulators in mammals. Mol Cell Biol 25: 10005–10016. 16260614 10.1128/MCB.25.22.10005-10016.2005 PMC1280273

[53] Jin Y, Suzuki H, Maegawa S, Endo H, Sugano S, et al (2003) A vertebrate RNA-binding protein Fox-1 regulates tissue-specific splicing via the pentanucleotide GCAUG. EMBO J 22: 905–912. 12574126 10.1093/emboj/cdg089 PMC145449

[54] Damianov A, Black DL (2010) Autoregulation of Fox protein expression to produce dominant negative splicing factors. RNA 16: 405–416. 20042473 10.1261/rna.1838210 PMC2811669

[55] Kiehl TR, Shibata H, Vo T, Huynh DP, Pulst SM (2001) Identification and expression of a mouse ortholog of A2BP1. Mamm Genome 12: 595–601. 11471052 10.1007/s00335-001-2056-4

[56] Gallagher TL, Arribere JA, Geurts PA, Exner CR, McDonald KL, et al (2011) Rbfox-regulated alternative splicing is critical for zebrafish cardiac and skeletal muscle functions. Dev Biol 359: 251–261. 21925157 10.1016/j.ydbio.2011.08.025 PMC3521583

[57] Gehman LT, Stoilov P, Maguire J, Damianov A, Lin CH, et al (2011) The splicing regulator Rbfox1 (A2BP1) controls neuronal excitation in the mammalian brain. Nat Genet 43: 706–711. 21623373 10.1038/ng.841 PMC3125461

[58] Howell VM, de Haan G, Bergren S, Jones JM, Culiat CT, et al (2008) A targeted deleterious allele of the splicing factor SCNM1 in the mouse. Genetics 180: 1419–1427. 18791226 10.1534/genetics.108.094227 PMC2581945

[59] Howell VM, Jones JM, Bergren SK, Li L, Billi AC, et al (2007) Evidence for a direct role of the disease modifier SCNM1 in splicing. Hum Mol Genet 16: 2506–2516. 17656373 10.1093/hmg/ddm206

[60] Zhang Q, Bethmann C, Worth NF, Davies JD, Wasner C, et al (2007) Nesprin-1 and -2 are involved in the pathogenesis of Emery Dreifuss muscular dystrophy and are critical for nuclear envelope integrity. Hum Mol Genet 16: 2816–2833. 17761684 10.1093/hmg/ddm238

[61] Li Z, Takakura N, Oike Y, Imanaka T, Araki K, et al (2003) Defective smooth muscle development in qkI-deficient mice. Dev Growth Differ 45: 449–462. 14706070 10.1111/j.1440-169x.2003.00712.x

[62] Zhao L, Ku L, Chen Y, Xia M, LoPresti P, et al (2006) QKI binds MAP1B mRNA and enhances MAP1B expression during oligodendrocyte development. Mol Biol Cell 17: 4179–4186. 16855020 10.1091/mbc.E06-04-0355 PMC1635361

[63] Hall MP, Nagel RJ, Fagg WS, Shiue L, Cline MS, et al (2013) Quaking and PTB control overlapping splicing regulatory networks during muscle cell differentiation. RNA 19: 627–638. 23525800 10.1261/rna.038422.113 PMC3677278

[64] Pistoni M, Shiue L, Cline MS, Bortolanza S, Neguembor MV, et al (2013) Rbfox1 downregulation and altered calpain 3 splicing by FRG1 in a mouse model of Facioscapulohumeral muscular dystrophy (FSHD). PLoS Genet 9: e1003186. 23300487 10.1371/journal.pgen.1003186 PMC3536703

[65] Venables JP, Brosseau JP, Gadea G, Klinck R, Prinos P, et al (2013) RBFOX2 is an important regulator of mesenchymal tissue-specific splicing in both normal and cancer tissues. Mol Cell Biol 33: 396–405. 23149937 10.1128/MCB.01174-12 PMC3554129

[66] Bland CS, Wang ET, Vu A, David MP, Castle JC, et al (2010) Global regulation of alternative splicing during myogenic differentiation. Nucleic Acids Res 38: 7651–7664. 20634200 10.1093/nar/gkq614 PMC2995044

[67] Venables JP, Lapasset L, Gadea G, Fort P, Klinck R, et al (2013) MBNL1 and RBFOX2 cooperate to establish a splicing programme involved in pluripotent stem cell differentiation. Nat Commun 4: 2480. 24048253 10.1038/ncomms3480

[68] Sergeant N, Sablonniere B, Schraen-Maschke S, Ghestem A, Maurage CA, et al (2001) Dysregulation of human brain microtubule-associated tau mRNA maturation in myotonic dystrophy type 1. Hum Mol Genet 10: 2143–2155. 11590131 10.1093/hmg/10.19.2143

[69] Dhaenens CM, Schraen-Maschke S, Tran H, Vingtdeux V, Ghanem D, et al (2008) Overexpression of MBNL1 fetal isoforms and modified splicing of Tau in the DM1 brain: two individual consequences of CUG trinucleotide repeats. Exp Neurol 210: 467–478. 18177861 10.1016/j.expneurol.2007.11.020

[70] Carpentier C, Ghanem D, Fernandez-Gomez FJ, Jumeau F, Philippe JV, et al (2014) Tau exon 2 responsive elements deregulated in myotonic dystrophy type I are proximal to exon 2 and synergistically regulated by MBNL1 and MBNL2. Biochim Biophys Acta 1842: 654–664. 24440524 10.1016/j.bbadis.2014.01.004

