# Supplementary figures and images for: RBFOX1 Cooperates with MBNL1 to Control Splicing in Muscle, Including Events Altered in Myotonic Dystrophy Type 1
Source: PLoS One. 2014 Sep 11;9(9):e107324. doi: 10.1371/journal.pone.0107324 (PMC4161394; doi:10.1371/journal.pone.0107324)

**A**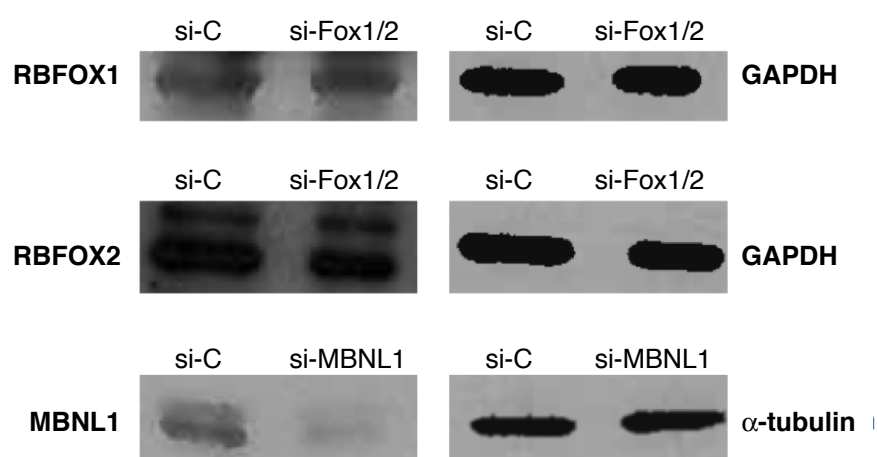**B**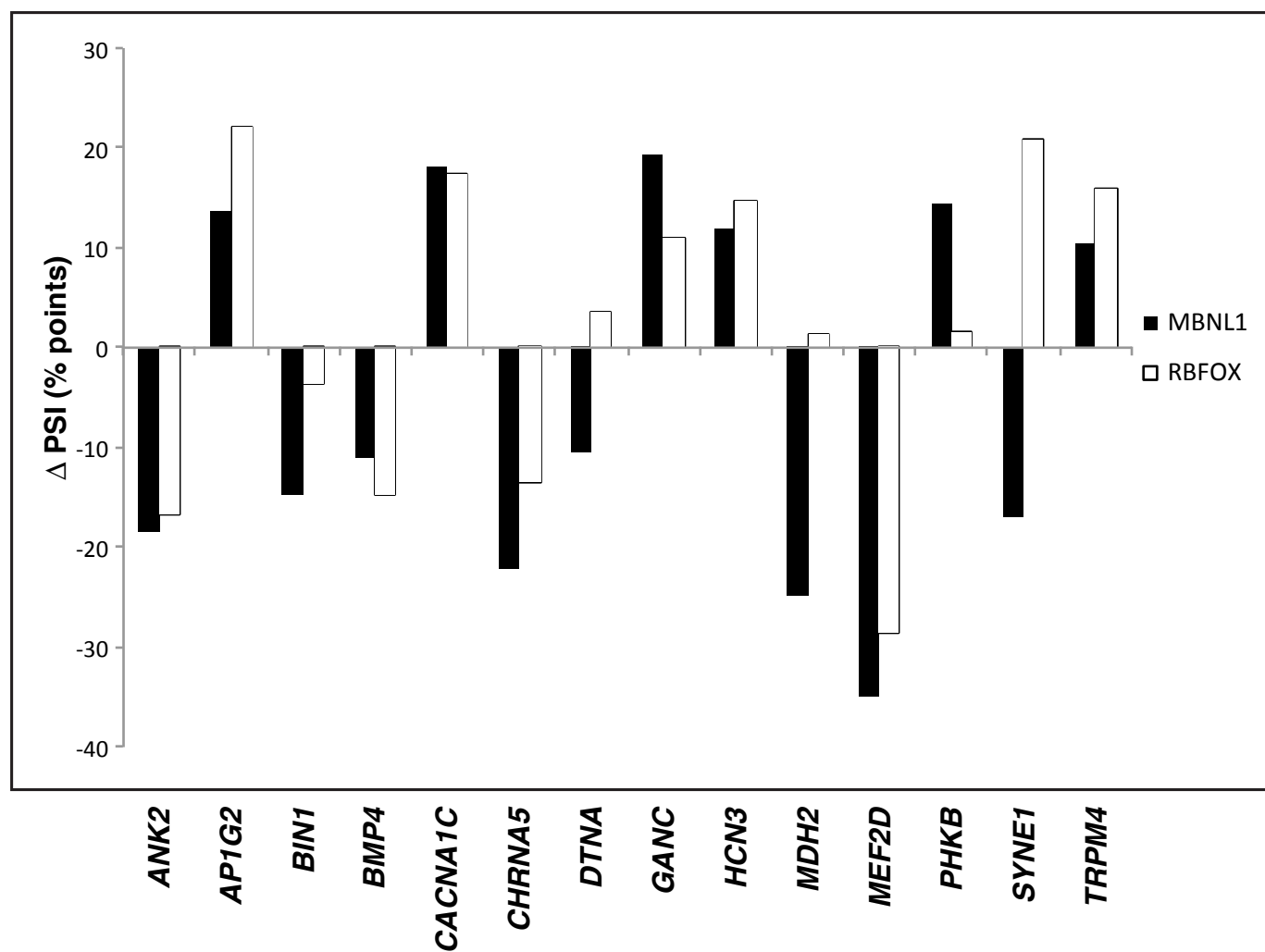

Supplementary Figure 1

Supplement: Figure S1 — ASEs that are co-regulated by MBNL1 and RBFOX1/RBFOX2. A. Immunoblot analysis following the knockdown of RBFOX1/RBFOX2 and MBNL1 in a HFN cell culture. A control siRNA (si-C) was also used, and housekeeping proteins (GAPDH or α-tubulin) were tested as loading controls. B. Histograms showing splicing changes (ΔΨ) observed for ASEs following MBNL1 (black bars) or RBFOX1/2 (white bars) knockdown, relative to si-C-treated HFN cells. (PDF) [file pone.0107324.s001.pdf]

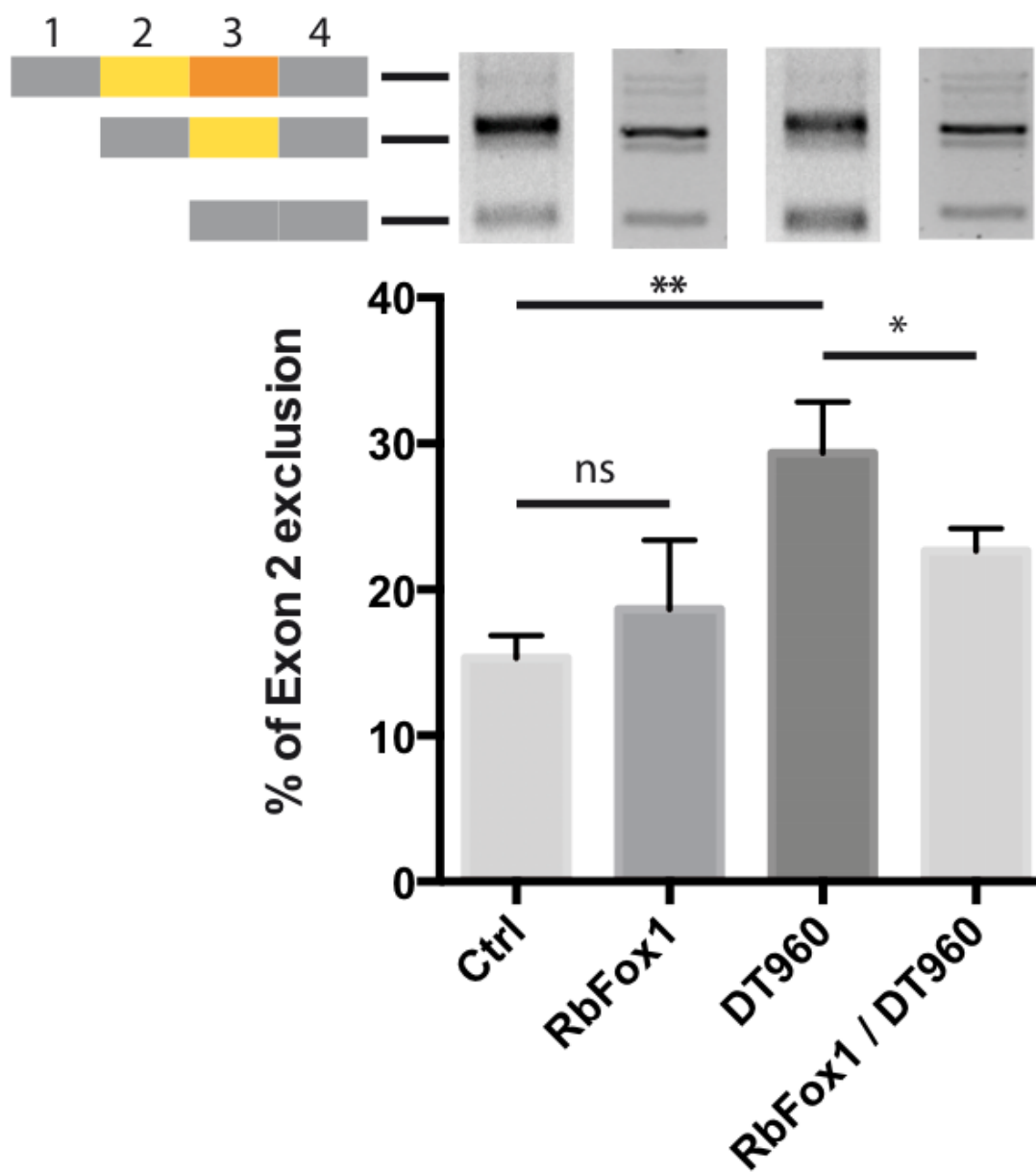

Supplementary Figure 2

Supplement: Figure S2 — RBFOX1 represses the impact of CUG-repeats on tau splicing in human glioblastoma T98G cells. T98G cells were transfected with expression vectors for RBFOX1, CUG-repeats (DT960) or both. Agarose gel of RT-PCR reactions designed to amplify tau splicing products are shown on top, and histograms depict exon 2 exclusion level in percentage with standard deviations. * = p<0.05; ** = p<0.01. In non-DM1 mimicking conditions, RBFOX1 did not significantly modify the splicing of tau exon 2. DT960 increased tau exon 2 exclusion, and this effect was partially prevented by co-expressing RBFOX1. (PDF) [file pone.0107324.s002.pdf]
