# Supplementary material for: RBFOX1 Cooperates with MBNL1 to Control Splicing in Muscle, Including Events Altered in Myotonic Dystrophy Type 1
Source: PLoS One. 2014 Sep 11;9(9):e107324. doi: 10.1371/journal.pone.0107324 (PMC4161394; doi:10.1371/journal.pone.0107324)
Supplement: Text S1 — Supplementary material and methods. (DOCX) [file pone.0107324.s005.docx]

**SUPPLEMENTARY MATERIAL AND METHODS**

**Tau splicing.** The human glioblastoma T98G (T98) cell line was maintained in DMEM medium supplemented with 10% fetal calf serum, 50 IU/ml of penicillin, 50 mg/ml of streptomycin and 4 mmol/L of glutamine. T98 cells were co-transfected with DT960 or pcDNA3.1 empty vector as a control. Co-transfections were achieved using the Fugene HD transfection reagent (Roche). For each well of a 12 well-plaque, we used 1 µg of DNA for each 3 µL of transfectant reagent. A total of 2 µg of plasmid was transfected with a 1:1 ratio for each vector. The *RbFox1* cDNA was subcloned in the pcDNA 3.1 expression vector (kindly provided by N. Charlet-Berguerand, IGBMC, Strasbourg, France). The DT960 plasmid (kindly provided by Tom Cooper, Houston, USA) contains the equivalent of 960 CTG repeats interrupted by a CTCGA motif at every 20 CTG, and was used to mimic the DM mutation [[1](#_ENREF_2)]. Noteworthy, the CTCGA interruption motif is different from the canonical (U)GCAUG RBFOX1 binding motif.

Total RNA was isolated 24 hours post-transfection using the Nucleospin® RNA II kit (Macherey Nagel, Düren, Germany). RNA concentration was determined using Nanodrop ND1000 (Labtech, France). RT-PCR was carried out in at least in three independent experiments. A total of 1.5 µg of RNA was reverse transcribed using random hexamers (5 µM/L) with the M-MLV reverse transcriptase (Invitrogen, California, USA) according to standard protocols. No DNA amplification was observed in control assays that lacked RNA. PCR was carried out in a final volume of 10 µL, with 10 pM of each primer under the following conditions, 5 µL of GoTaq® GreenMasterMix and 2 µL of Nuclease free H20 (Promega): 3 min at 94°C, 32 cycles of a 30 sec denaturation step at 94°C, annealing for 1 min at specific temperature, 1 min of extension and 7 min of final extension at 72°C. For Tau exon 2/3 amplication, the following PCR primers were used : Forward primer 5’-TACGGGTTGGGGGACAGGAAAGAT-3’ and reverse primer 5’-GGGGTGTCTCCAATGCCCTGCTTCT-3’. 18S rRNA was used as an internal control. Reaction products were resolved by electrophoresis using a 3% agarose gel, and bands were stained with BET (Invitrogen). The intensity of BET luminescence was measured using a Molecular Imager® Gel Doc TM XR System (Bio-Rad).

For the statistical analysis, the values were reported as the mean ± SEM for at least three independent experiments (GraphPad Software Inc., San Diego, USA). An ANOVA-Two test was used to compare groups.

**Supplementary Reference**

1. Philips AV, Timchenko LT, Cooper TA (1998) Disruption of splicing regulated by a CUG-binding protein in myotonic dystrophy. Science 280: 737-741.
